# From participants to partners: a strategy for establishing a national consumer fellow to lead the co-design of maternity patient-reported experience measures (PREMs) and patient-reported outcome measures (PROMs)

**DOI:** 10.1186/s41687-026-01065-6

**Published:** 2026-04-13

**Authors:** Karen Schlage, Lauren C. Spark, Emily J. Callander, Emily Leefhelm, Zoe Bradfield

**Affiliations:** 1 School of Nursing, Curtin University, Perth, WA Australia; 2 School of Public Health, University of Technology Sydney, Sydney, NSW Australia; 3 Monash Centre for Health Research and Implementation, Monash University, Melbourne, VIC Australia; 4 Royal Prince Alfred Hospital, Sydney, NSW Australia; 5 Women and Newborn Health Service, North Metropolitan Health, Perth, WA Australia

**Keywords:** Maternity care experiences, Maternity care outcomes, Measurement tools, Patient-reported measures, PREMs, PROMs, Patient engagement, Co-design, Consumer

## Abstract

**Background:**

Patient‑reported experience measures (PREMs) and patient‑reported outcome measures (PROMs) play an increasingly important role in assessing the quality and safety of healthcare. However, their application in maternity care is relatively contemporary, and progress is hindered by the absence of high‑quality, validated, maternity‑specific tools. In Australia, there are no nationally standardized maternity PREMs or PROMs capable of capturing whether care provided during the transformative period of childbearing aligns with the needs, expectations, and priorities of those giving birth. Without genuine consumer co‑design, current approaches to developing maternity PREMs and PROMs risk failing to capture the complexity and diversity of women’s experiences and outcomes.

**Main text:**

The development of national consumer co-designed maternity PREMs and PROMs in Australia is a critical opportunity to ensure that which is measured, genuinely reflects what matters to women. Globally however, maternity care lacks validated PREMs and PROMs that have been meaningfully co‑designed with consumers. Addressing this gap requires intentional, rigorous co‑design grounded in five core principles: authentic consumer leadership; diverse and inclusive representation; iterative, continuous engagement; respectful and reciprocal partnership supported by equitable reimbursement; and alignment with best‑practice frameworks that safeguard against tokenism. Together, these principles underpin contemporary models of consumer involvement, including dedicated consumer leadership roles and structured advisory groups, to meaningfully embed lived experience to co-design maternity care PREMs and PROMs.

**Conclusions:**

A co-design approach that centers women as active partners, rather than passive participants, is essential for producing PREMs and PROMs that are relevant, acceptable and impactful in real-world maternity care. Rigorous, inclusive co‑design provides the foundation for measurement systems capable of driving meaningful and lasting improvements in the safety, quality, and person‑centeredness of maternity care.

## Background

Alongside ‘traditional’ measures of clinical excellence in maternity care, such as low rates of death and morbidity, PREMs and PROMs can serve as indicators of safety, quality and high-value care [1]. Whilst PREMs and PROMs are well established in many areas of healthcare, their use in maternity care is comparatively recent. Two recent systematic reviews appraised the validity of existing maternity care PREMs and PROMs tools around the world. Authors reported most tools were founded on poor quality evidence, with little description of how women were involved in tool development, and sparse detail regarding evidence of content validity [2, 3].

While such tools are used internationally, Australia does not have any nationally standardized PREMs or PROMs to assess whether care during pregnancy, birth, and the early postnatal period aligns with the health needs and priorities identified by those giving birth.

Despite calls for the development and implementation of maternity PREMs and PROMs in the national maternity strategy and from industry leads [4], no validated tools are currently available for national use [5]. This absence leaves individual services, and more broadly, the health system unable to consistently understand, benchmark, or respond to women’s^1^ experiences and outcomes of care.

The need to address this gap is increasingly urgent. Without patient-reported data that authentically reflects women’s perspectives, Australian services or the system more broadly, cannot meaningfully evaluate or improve the safety and quality of maternity care. This is particularly problematic in the context of rising concerns about birth trauma, and care that fails to meet expectations [6]. PREMs and PROMs offer a mechanism to center women’s experiences at service, health district and national levels, supporting a maternity care system that is equitable, safe, patient-centered and high-quality [1].

However, for these measures to fulfil their purpose, they must be developed with methodological rigor and grounded in what women themselves identify as what they want and need from maternity care [3]. This requires genuine commitment to, and sustained engagement in, co-design principles with maternity care consumers, healthcare professionals and policymakers. Understanding which experiences and outcomes ‘matter most’ to women is essential for driving meaningful improvements to enhance the safety, quality, and lived experience of maternity care in Australia.

## Main text

A current Australian initiative, the Measuring what Matters to Australian Mothers (MMAMs) study, highlights a national effort to develop and validate consumer co-designed maternity care PREMs and PROMs that reflect the priorities of those who give birth [7]. This initiative underscores the growing recognition that the measurement and benchmarking of safety and quality of maternity care in Australia must be driven by what matters most to women. A number of consumer co-designed guiding principles have underpinned the MMAMs study, forming the foundation of a rigorous and authentic co-design approach that centers women’s lived experience, genuine influence, and strong ongoing collaboration throughout the research process. The next section will highlight five key consumer-grounded principles crucial for elevating consumer co-design to capture, illuminate, and drive the development of patient-reported measures to meaningfully improve the safety and quality of maternity care in Australia.

### Principles of rigorous consumer co-design

#### Authentic consumer leadership

Rigorous co‑design begins with authentic consumer leadership, where people with lived experience are not only invited into the process but hold meaningful influence within it. Co‑design requires structures that enable consumers to shape priorities, contribute expertise, and guide decisions in ways that avoid tokenism and elevate lived experience as a critical source of knowledge. One way to support this is through formal appointment of a paid consumer leadership position such as a Consumer Fellow or equivalent, with dedicated project funding at a level commensurate with other core research or academic roles. The application of a role title is an important step in recognizing the intentional and distinctive elevation of Consumer participation as an integrated part of the investigator team. Dedicated remuneration recognizes the legitimacy and value of lived‑experience expertise, ensures equity of contribution, and embeds consumer leadership as a sustained and respected element of the project’s governance. Such a role may include chairing consumer advisory groups, representing the consumer perspective in system‑level discussions, and advocating for consumer‑informed input across all aspects of design and decision‑making. Recruitment to this position should be intentional, seeking individuals who can empower diverse consumer voices, support consensus‑building, and bring a thoughtful, pragmatic lens to how lived experience informs project objectives.

#### Diverse and inclusive representation

Lived experience should be positioned at the core of any genuine co‑design approach. One way to embed this expertise meaningfully is through a structured governance mechanism, such as an Expert Consumer Advisory Group established to provide a formal, consistent avenue for consumer influence. Membership should be intentionally recruited through an expression‑of‑interest process to ensure broad and diverse representation across demographic characteristics, including age, gender identity, varied maternity care experiences and outcomes, cultural background, and socioeconomic context. Importantly, consumers should be fairly remunerated for their time, expertise, and contribution. Providing honorariums or payment acknowledges lived experience as legitimate expertise, reinforces equity between consumers and professional contributors, and reduces financial barriers that might otherwise limit deep and prolonged project participation. Bringing together people with diverse perspectives enriches the development process, strengthens equity and relevance, and ensures that the resulting tools respond to the full spectrum of maternity care experiences and priorities.

#### Iterative and continuous engagement

Co‑design is not a single consultation, but a sustained and intentionally structured process. A rigorous approach requires repeated opportunities for consumers to shape the work as it evolves, ensuring their perspectives influence each stage of development. This includes engaging consumers during conceptualization, refinement, and testing, allowing their insights to guide both the direction and integrity of the work. Mixed‑method processes such as e‑Delphi rounds that invite iterative prioritization, alongside qualitative methods like focus group interviews, provide complementary avenues for consumers to articulate, deepen, and clarify their contributions. Integrating both quantitative and qualitative insights, and returning to consumers at multiple time points, keeps the process grounded in lived experience and ensures that the tools developed remain relevant, responsive, and fit for purpose.

#### Foundations of trust, respect, and reciprocity

A rigorous co‑design approach is grounded in clear expectations, transparent communication, and a commitment to mutual benefit. At its core is a respectful recognition that lived experience is a form of expertise, deserving of the same legitimacy and value as professional or academic knowledge. Equitable reimbursement and therefore adequate dedicated project funding to support this component is essential in this context. Prioritizing respectful relationships, fairness, and shared understanding creates the conditions in which people feel safe, valued, and able to contribute meaningfully. These elements are fundamental to genuine partnership and ultimately determine whether co‑design processes produce outcomes that are trustworthy, equitable, and responsive to women’s needs.

#### Alignment with best-practice frameworks

A robust co‑design approach must be anchored in established best‑practice frameworks that articulate the values, structures, and expectations necessary for meaningful engagement. Guidance, such as the World Health Organization (WHO) lived‑experience principles [8], and Consensus‑based Standards for the Selection of Health Measurement Instruments (COSMIN) standards for measurement design [9], provides a shared language and evidence‑based foundation for equitable, transparent, and accountable co‑design. These frameworks help ensure that power is shared rather than assumed, that accessibility and inclusion are intentionally planned for, and that consumer input meaningfully shapes decisions rather than being symbolic. Aligning co‑design processes with established standards strengthens methodological integrity, clarifies expectations for all contributors, and provides structures that help safeguard against tokenism. Importantly, these frameworks support the development of tools that are credible and rigorously constructed.

## Conclusions

Taken together, these principles demonstrate that rigorous consumer co‑design is not simply a methodological preference but a fundamental requirement for developing maternity PREMs and PROMs that are credible, equitable, and genuinely reflective of women’s perspectives and experiences. When lived experience is centered through authentic leadership, diverse representation, sustained engagement, respectful partnership, and alignment with best‑practice frameworks, the resulting measures are far more likely to capture what truly matters to those who use maternity services. This depth of partnership moves co‑design beyond consultation toward genuine collaboration, ensuring that the tools developed are not only methodologically sound but grounded in the experiences, priorities, and values of the women they are designed to serve. In doing so, consumer co‑design becomes a powerful mechanism for shaping maternity care measurement tools that can drive meaningful, lasting improvements in safety, quality, and person‑centered care.

## Data Availability

There are no primary data available for this commentary.

## Footnotes

1 The terms “woman” and “women” are used inclusive of people who do not identify as women but who are pregnant or have given birth.

## Abbreviations

PREMs: Patient-Reported Experience Measures
PROMs: Patient-Reported Outcome Measures
